# Renal Angina Indices and Urinary Biomarkers: A Combined Approach to Predict Acute Kidney Injury in Critically Ill Patients

**DOI:** 10.34067/KID.0000001020

**Published:** 2025-11-12

**Authors:** Erick Y. Zúñiga-González, Karla I. Linares-Robles, Juan M. Villegas-Gamas, Noemí Del Toro-Cisneros, Cristino Cruz-Rivera, María José López-Ruelas, Rosario G. Hernández-Ortega, Pablo Galindo, Yazmin A. Mercado-Hernández, Olynka Vega-Vega

**Affiliations:** 1 Department of Nephrology and Mineral Metabolism, Instituto Nacional de Ciencias Médicas y Nutrición Salvador Zubirán, Mexico City, Mexico; 2 Department of Nephrology, Instituto de Seguridad Social del Estado de México y Municipios, Mexico City, Mexico; 3 Department of Nephrology, Hospital General de México, Mexico City, Mexico

**Keywords:** AKI, biomarkers

## Abstract

**Key Points:**

- The modified renal angina index outperformed prior indices for early severe AKI prediction in critically ill adults.
- Combining modified renal angina index with [tissue inhibitor of metalloproteinase-2]×[IGF binding protein 7] improved area under the receiver operating characteristic curve, although not statistically significant, requiring external validation.
- Early AKI detection supports timely nephroprotective actions and individualized management in critical care.

**Background:**

Several renal angina indices (RAIs) have been developed to stratify AKI risk in critically ill populations. However, limited evidence supports whether incorporating urinary biomarkers enhances predictive performance. This study aimed to evaluate the diagnostic accuracy of three RAIs, with and without urinary biomarkers integration, for predicting severe AKI (defined as Kidney Disease Improving Global Outcomes stages 2–3) in critically ill adults.

**Methods:**

In this prospective, multicenter diagnostic study, we assessed the ability of three RAIs to predict severe AKI, defined as Kidney Disease Improving Global Outcomes stages 2–3, within 72 hours of intensive care unit admission. We further evaluated whether the addition of urinary biomarkers improve predictive accuracy, using area under the receiver operating characteristic curve (AUC) and net reclassification improvement (NRI).

**Results:**

We enrolled 134 critically ill patients, of whom 15% developed severe AKI within 72 hours. Diagnostic performance of RAIs and urinary biomarkers alone was modest (AUC: Matsuura RAI, 0.63; Del Toro RAI, 0.71; and modified RAI [mRAI], 0.76). Incorporating urinary biomarkers improves the predictive performance of all RAIs, particularly enhancing specificity. Among biomarkers assessed, (tissue inhibitor of metalloproteinase-2 [TIMP‐2])×(IGF binding protein 7 [IGFBP7]) demonstrated the greatest capacity to reclassify patients toward event prediction across RAI models. The combination of mRAI with [TIMP‐2]×[IGFBP7] yields the highest absolute predictive performance (AUC, 0.82; 95% confidence interval, 0.72 to 0.93).

**Conclusions:**

Sequential risk stratification combining clinical RAIs with targeted urinary biomarkers profiling significantly improves early prediction of severe AKI in critically ill adults' patients. Although RAIs and urinary biomarkers alone show limited predictive accuracy, their integration—particularly mRAI plus [TIMP‐2]×[IGFBP7]—optimizes discrimination and may facilitate earlier intervention in high-risk intensive care unit adult patients.

## Visual Abstract

**Figure:**
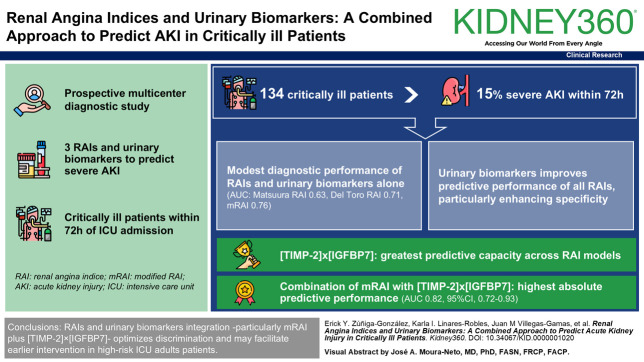

## Introduction

AKI affects 50%–60% of critically ill patients in intensive care units (ICUs) and is associated with worse outcomes, particularly in those requiring KRT.^1–3^ Early diagnosis and timely interventions are essential to improve patient outcomes. However, current diagnostic criteria based on the 2012 Kidney Disease Improving Global Outcomes (KDIGO)^4^ guidelines using serum creatinine (SCr) levels and urine output demonstrate limited sensitivity and specificity for the early detection of AKI.^5,6^ Accurate risk prediction offers a crucial window for preventive strategies and close monitoring during the subclinical phase, potentially mitigating progression.^7^ Clinical prediction models, such as renal angina index (RAI)^8,9^ and urinary biomarker panels, have emerged as promising tools to improve early AKI risk assessment.^6,9^

The RAI, analogous to angina pectoris in acute coronary syndrome, integrates early functional changes with clinical risk factors to raise suspicion for AKI.^6,10^ While highly sensitive with excellent negative predictive value (NPV) in pediatric cohorts, its performance in adults has been modest despite various adaptations. Efforts are ongoing to refine RAI models for critically ill adult populations.

Novel urinary biomarkers detect injury across different nephron segments and often outperform early SCr changes,^5,11–13^ with reported areas under the receiver operating characteristic curve (AUC) of 0.8–0.9 in diverse ICU cohorts.^11–13^ However, even the most extensively studied urinary biomarkers have exhibited inconsistent performance depending on the clinical context and patient population, which has limited their widespread adoption in routine clinical practice. Like troponin I, which has limited specificity for acute coronary syndrome in patients without chest pain,^14^ AKI biomarkers are most useful when applied in high pretest probability settings, supporting context-guided integration with clinical risk models.

The objective was to evaluate the predictive performance of three RAIs models (Matsuura *et al.*,^6^ Del-Toro-Cisneros *et al.*,^8^ and a RAI derived from this cohort modified RAI [mRAI]), both with and without the integration of urinary biomarkers, for early detection of severe AKI (KDIGO stage 2 or 3) in critically ill adults from two tertiary care hospitals in Mexico.

## Methods

### Study Population

This was a prospective, multicenter, and observational study conducted in two ICUs located in distinct tertiary care hospitals in Mexico. The study population consisted exclusively of Mexican ethnicity. The study was performed in accordance with the principles outlined in the Declaration of Helsinki and received ethical approval from the Institutional Review Boards for the Instituto Nacional de Ciencias Médicas y Nutrición Salvador Zubirán, México (approval number NMM-4223), and Centro Medico ISSEMYM Ecatepec, Mexico (approval number PICME-2023-29). Written informed consent was obtained from all patients or their legal representatives.

Patient enrollment took place between August 2022 and March 2024, including individuals admitted to the ICU during this period. Exclusion criteria were as follows: (*1*) younger than 18 years, (*2*) AKI at or before ICU admission, (*3*) known history of CKD (eGFR <45 ml/min per 1.73 m^2^), or (*4*) prior kidney transplantation. Patients who died or were discharged within 48 hours after enrollment were excluded. All participants received standardized organ support and treatment based on KDIGO guidelines.^4^

Baseline data included demographics, admission diagnosis, comorbidities, anthropometrics, laboratory parameters, and severity scores (Acute Physiology and Chronic Health disease Classification System II, nonrenal Sequential Organ Failure Assessment [SOFA], Charlson Index). The use of vasopressors/inotropes and mechanical ventilation (MV) was recorded. SCr was measured at ICU admission; baseline SCr was defined as the mean value from the preceding 6 months or, if unavailable, the lowest in-hospital value (applied in 20/134 patients [15%]).

### Calculated Variables and RAIs

**Figure 1:**
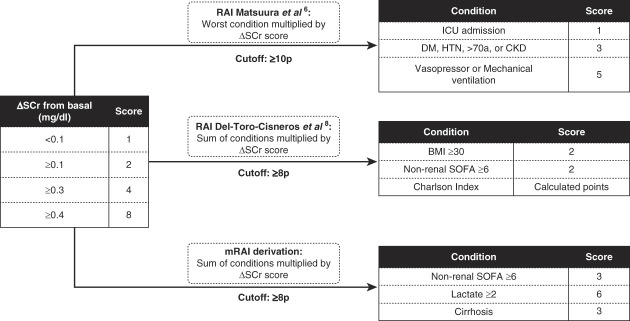
**RAIs.** Three different scores were used for calculation of RAI. Two from previous described indices and the one derived in this population. BMI, body mass index; DM, diabetes mellitus; HTN, hypertension; ICU, intensive care unit; mRAI, modified renal angina index; RAI, renal angina index; ΔSCr, change in serum creatinine; SOFA, Sequential Organ Failure Assessment.

The RAI derived from this cohort was calculated at ICU admission as a composite AKI risk score. Candidate variables were selected using logistic regression (Supplemental Table 1), and the total score was computed as the sum of individual variable scores. Renal angina was defined at >8 points (Supplemental Table 2). Two previously validated RAI models were evaluated: Matsuura *et al.*^6^ and Del-Toro-Cisneros *et al*.^8^ All RAIs incorporated a change in SCr (ΔSCr) score as follows: ≥0.4 mg/dl=8 points; ≥0.3 mg/dl=4 points; ≥0.1 mg/dl=2 points; <0.1 mg/dl=1 point (Figure 1).

Matsuura RAI: five points for MV and/or vasopressor use; three points for ≥1 comorbidity (diabetes mellitus, age >70 years, CKD, or hypertension); and one point for ICU admission. The final score was the highest condition score multiplied by the ΔSCr score. Renal angina was fulfilled at ≥10 points.

Del-Toro-Cisneros RAI: two points for nonrenal SOFA ≥6; two points for body mass index ≥30 kg/m^2^; plus the Charlson index score. The total was multiplied by the ΔSCr score (same categories as above). Renal angina was fulfilled at ≥8 points.

The presence or absence of renal angina was denoted as “RAI+” or “RAI−,” respectively.

### Biomarker Measurements

Urine samples were collected within 24 hours of ICU admission, centrifuged at 3000 rpm for 10 minutes at 4°C, and supernatants stored at −80°C. Biomarkers were quantified using commercially available, human-specific ELISAs as follows: neutrophil gelatinase-associated lipocalin, C–C motif chemokine ligand 14, kidney injury molecule-1, IGF binding protein 7 (IGFBP7), tissue inhibitor of metalloproteinase-2 (TIMP-2; DY1757, DY1578, DKM100, DY1334-05, DY971, R&D Systems), and heat shock protein 72 (MBS705388, MyBioSource). Assays were validated in triplicate with representative samples (no AKI, AKI stages 1–3) to assess detection capacity and intra-assay variability. Patient samples were then measured once per run with standard curves and quality controls. Laboratory staff were blinded to clinical data. Optimal cutoffs for AKI prediction were determined using sensitivity analysis and Youden index.

### Outcomes and Definitions

The diagnosis of AKI was based exclusively on SCr criteria according to the KDIGO guidelines^4^: (*1*) SCr ≥0.3 mg/dl (≥26.5 *μ*mol/L) within 48 hours, (*2*) increase in SCr to ≥1.5 times baseline, and (*3*) SCr ≥3× times baseline or SCr >4 mg/dl or KRT initiation. The primary end point was the development of severe AKI (KDIGO stage 2 or 3) within 72 hours of the ICU admission, consistent with previous RAI studies.^15,16^ These stages were selected given their strong association with dialysis initiation and mortality. Although this cutoff may underestimate the incidence of AKI occurring beyond 72 hours, it was selected to enhance the clinical relevance of early risk stratification.

In an exploratory *post hoc* analysis, we also evaluated the occurrence of acute kidney disease (AKD), defined according to KDIGO criteria as persistent kidney dysfunction beyond 7 days and up to 90 days after AKI onset. Operationally, AKD was defined as an eGFR <60 ml/min per 1.73 m^2^ or a ≥35% decline from baseline kidney function.

### Statistical Analysis

The distribution of continuous variables was assessed by using the Kolmogorov-Smirnov test. Descriptive statistics are expressed as numbers (percentages) and medians (interquartile ranges) or mean (SD), as appropriate. Baseline patient characteristics between patients with or without AKI were analyzed using the Mann–Whitney *U* test. Chi-square of Fisher exact tests were used for categorical variables.

Predictive performance of RAIs and urinary biomarkers for severe AKI was evaluated using receiver-operating characteristic analysis. Severe AKI was predicted using simple logistic regression for each RAI and biomarker and multivariable models combining RAIs with biomarkers. AUC/(C-statistic) was calculated for each prediction model (RAI and biomarker concentrations used as continuous variables) and compared using DeLong method.^17^ Performance improvement of the prediction models of RAI+ biomarkers were also evaluated by the Akaike information criterion, net reclassification improvement (NRI), and integrated discrimination improvement.^18,19^ No risk categories were selected for the calculation of NRI. Decision-trees were constructed for each RAI and urinary biomarker (Supplemental Figure 1), categorizing patients based on predefined decision rules. Probability of AKI was calculated by rate of AKI per subpopulation of RAI and biomarkers combination.

To address potential bias from missing biomarker data (12% of the cohort), we performed a sensitivity analysis using multiple imputation with predictive mean matching (20 imputations). The imputation model included all variables from the main analyses, urinary biomarkers, and the outcome (severe AKI). Pooled estimates across imputations were calculated using Rubin rules (Supplemental Table 5).

Formal *a priori* sample size calculation was not performed; however, the final sample of 134 patients with 20 severe AKI provided >80% power to detect AUCs ≥0.75 with a two-sided α of 0.05 (Hanley-McNeil approach^20^) consistent with the effect sizes observed.

Two-sided *P* < 0.05 was considered significant. All analyses were performed using Statistical Package for the Social Sciences 25.0 (IBM, Armonk, NY) and GraphPad Prism 10.0 (Boston, MA).

## Results

### Overall Patient Characteristics and Outcomes

A total of 134 patients were included in the final analysis. Of these, 20 (15%) developed the primary outcome within 72 hours. Baseline characteristics are presented in Table 1. Sepsis was the leading cause of ICU admission. Patients who developed severe AKI exhibited higher SOFA scores, greater vasopressor requirements, and higher prevalence of cirrhosis.

**Table 1:** Baseline characteristics of patients at intensive care unit admission

Characteristic	Total (*N*=134)	No AKI or KDIGO 1 (*n*=114)	AKI KDIGO ≥2 (*n*=20)	*P* Value
Age, mean (SD)	53 (19)	53 (20)	54 (13)	0.68
Male, No. (%)	75 (56)	63 (55)	12 (60)	0.80
Weight, kg, median (IQR)	67 (59–80)	67 (58–78)	76 (60–84)	0.57
BMI, kg/m^2^, median (IQR)	25 (22–30)	25 (22–29)	27 (23–32)	0.31
**Primary reason for admittance to the ICU, *n* (%)**				
Sepsis	42 (31)	33 (29)	9 (45)	0.19
Circulatory dysfunction	31 (23)	28 (24.5)	3 (15)	0.56
CNS	30 (22)	28 (24.5)	2 (10)	0.24
Postoperative	27 (20)	22 (19)	5 (25)	0.55
Endocrine	1 (0.7)	1 (1)	0	1.00
Liver failure	3 (2.2)	2 (2)	1 (5)	0.38
SOFA score, median (IQR)	6 (3–19)	6 (3–10)	8 (6–11)	0.01
Charlson index, median (IQR)	3 (1–5)	3 (1–5)	3 (2–5)	0.36
**Comorbidities, No. (%)**				
Diabetes	35 (26)	29 (25)	6 (30)	0.78
Cirrhosis	15 (11)	9 (8)	6 (30)	0.01
Rheumatologic	19 (14)	15 (13)	4 (20)	0.48
Heart disease	50 (37)	43 (37)	7 (35)	1.00
Malignancy	14 (10)	11 (10)	3 (15)	0.43
Pulmonary disease	11 (8)	10 (9)	1 (5)	1.00
CKD	6 (4.5)	4 (3.5)	2 (10)	0.21
*Stage G2*		1 (0.9)	2 (10)	
*Stage G3A*		2 (1.8)		
Diuretic use	13 (19)	10 (9)	3 (15)	0.41
**Vasopressor support, No. (%)**	87 (65)	72 (63)	15 (75)	0.44
>1 vasopressor support	21 (16)	13 (11)	8 (40)	0.00
Ventilatory support, No. (%)	69 (51)	58 (51)	11 (55)	0.81
Balance fluid at admission, ml, median (IQR)	631 (−14 to 1552)	614 (−82 to 1496)	742 (106–2150)	0.88
**Laboratory at ICU admission**				
Hemoglobin, g/dl, median (IQR)	11.6 (9–14)	11.8 (9–14))	11.1 (8.5–14)	0.36
Albumin, g/dl, median (IQR)	3.3 (2.8–3.8)	3.4 (2.8–3.8)	3.1 (2.4–3.4)	0.03
Leukocytes, ×103/*µ*l, mean (SD)	10.2 (6.8–14)	10.05 (5.83)	15.8 (10.4)	0.33
Sodium, mEq/L, mean (SD)	139 (5.5)	139 (5.2)	138 (7.5)	0.42
Potassium, mEq/L, mean (SD)	3.9 (0.64)	3.9 (0.6)	4 (0.5)	0.70
Lactate, mmol/L, median (IQR)	1.5 (1–2.5)	1.5 (1–2.3)	2.7 (1.2–4.4)	0.01
Bicarbonate, mmol/L, mean (SD)	22.5 (4.8)	22.2 (5)	21 (4.2)	0.27
Total bilirubin, mg/dl, median (IQR)	0.8 (0.5–1.5)	0.7 (0.5–1.4)	1.2 (0.6–6.2)	0.04
ALT, U/L, median (IQR)	24 (15–45)	23 (14–42)	31 (19–68)	0.24
AST, U/L, median (IQR)	27 (17–54)	26 (16–48)	45 (24–76)	0.29
C-reactive protein, mg/dl, median (IQR)	8.5 (3–23)	7.7 (3–22)	14 (6–27)	0.73
Baseline SCr, mg/dl, mean (SD)	0.7 (0.24)	0.67 (0.24)	0.76 (0.23)	0.16
SCr at admission, mg/dl, mean (SD)	0.86 (0.33)	0.83 (0.33)	1 (0.26)	0.02
KRT, No. (%)	3 (2.2)	0 (0)	3 (15)	0.002
Mortality, No. (%)	24 (18)	21 (18)	3 (15)	1.00

ALT, alanine transaminase; AST, aspartate aminotransferase; BMI, body mass index; CNS, central nervous system; ICU, intensive care unit; IQR, interquartile range; KDIGO, Kidney Disease Improving Global Outcomes; SCr, serum creatinine; SOFA, Sequential Organ Failure Assessment.

### Factors Associated with AKI and mRAI Derivation

Univariate predictors of severe AKI included nonrenal SOFA ≥6, cirrhosis, dual vasopressors, elevated lactate, and bilirubin. In multivariable analysis (sex and age adjusted), nonrenal SOFA ≥6 (odds ratio [OR], 3.35; 95% confidence interval [CI], 0.94 to 11.88), cirrhosis (OR, 3.77; 95% CI, 0.93 to 15.3), and lactate >2 mmol/L (OR, 6.00; 95% CI, 1.86 to 19.5) remained independent predictors (Supplemental Table 1). These three variables were selected for the development of the mRAI (Table 2).

**Table 2:** Logistic regression analysis for development of modified renal angina index

Variable	Univariate		Multivariate	
	OR (95% CI)	*P* Value	ORa (95% CI)	*P* Value
Nonrenal SOFA ≥6	3.77 (1.27 to 11.17)	0.017	3.35 (0.94 to 11.88)	0.06
Cirrhosis	5.43 (1.66 to 17.7)	0.005	3.77 (0.93 to 15.3)	0.06
Double vasopressor	5.70 (1.94 to 6.773)	0.002		
Lactate >2 mmol/L	4.75 (1.63 to 13.89)	0.004	6 (1.86 to 19.5)	0.003
Bilirubin >2 mg/dl	5.03 (1.78 to 14.2)	0.002		

Logistic regression for univariate and multivariate analysis for severe AKI prediction. Variables with significant association in the univariate model are shown and included in a multivariate regression model, considering an entry criterion of *P* < 0.1 and removal criterion of *P* > 0.3. The model was adjusted for age and sex. CI, confidence interval; OR, odds ratio; ORa, adjusted odds ratio; SOFA, Sequential Organ Failure Assessment.

### Performance of RAIs in Predicting Severe AKI

RAI positivity was 56/134 (41.7%) for Matsuura, 63/134 (47.0%) for Del-Toro-Cisneros, and 55/134 (41.0%) for mRAI; 37/134 (27.6%) were positive on all three. Severe AKI among RAI(+) patients occurred in 21%, 22%, and 37%, respectively. All three RAIs predicted severe AKI (Matsuura RAI [OR, 1.05; 95% CI, 1.01 to 1.08]; Del-Toro-Cisneros RAI [OR, 1.03; 95% CI, 1.00 to 1.05]; mRAI [OR, 1.04; 95% CI, 1.02 to 1.07]), but none was associated with mortality or AKD.

**Figure 2:**
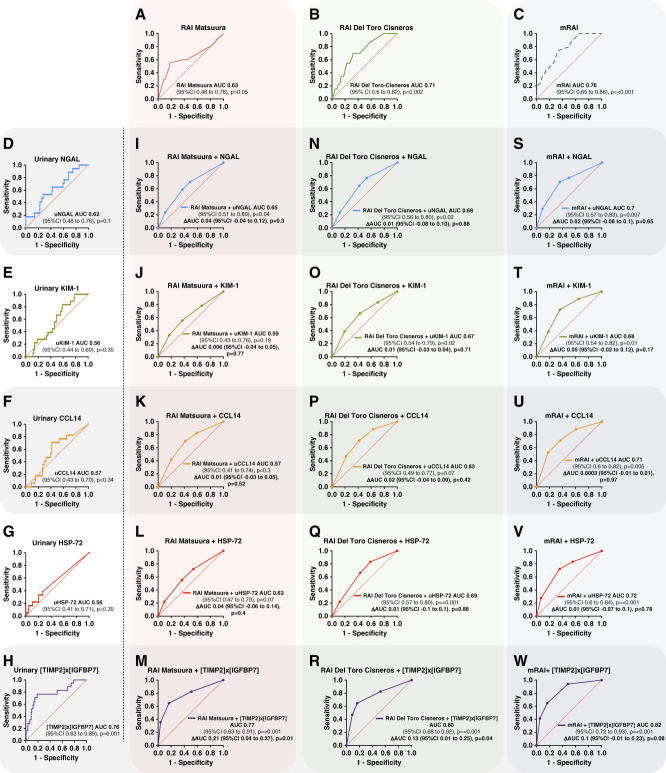
**ROC curves for prediction of severe AKI (<72 hours) in critically ill adults.** (A–C) Three clinical RAI models. (D–H) Individual urinary biomarkers. (I–W) Integrated models combining each RAI score with urinary biomarkers. The AUC and 95% CIs are shown in each panel, with changes in AUC indicated for the integrated models. AUC, area under the receiver operating characteristic curve; CCL14, C–C motif chemokine ligand 14; CI, confidence interval; HSP-72, heat shock protein 72; IGFBP7, IGF-binding protein 7; KIM-1, kidney injury molecule-1; NGAL, neutrophil gelatinase-associated lipocalin; ROC, receiver operating characteristic; TIMP2, tissue inhibitor of metalloproteinase-2; uCCL14, urinary C–C motif chemokine ligand 14; uHSP-72, urinary heat shock protein 72; uKIM-1, urinary kidney injury molecule–1; uNGAL, urinary neutrophil gelatinase–associated lipocalin.

The mRAI exhibited superior predictive performance, with AUC of 0.76 (95% CI, 0.65 to 0.86) and higher sensitivity and specificity compared with Del-Toro-Cisneros RAI (AUC, 0.71; 95% CI, 0.60 to 0.82). The Matsuura RAI showed the lowest predictive accuracy (Figure 2, A–C and Table 3).

**Table 3:** Predictive performance of renal angina indices and urinary biomarkers at admission for prediction of severe AKI

Index/Biomarker	AUC (95% CI)	*P* Value	Sensitivity (%) (95% CI)	Specificity (%) (95% CI)	PPV (%) (95% CI)	NPV (%) (95% CI)	LR+ (95% CI)	LR− (95% CI)
**Basal RAI performance**								
RAI Matsuura (≥10p)	0.63 (0.48 to 0.78)	0.05	60 (36 to 80)	61 (52 to 70)	15 (9 to 22)	89 (83 to 93)	1.55 (1 to 2.38)	0.65 (0.37 to 1.14)
RAI Del-Toro-Cisneros (≥8p)	0.71 (0.60 to 0.82)	0.002	70 (45 to 88)	57 (47 to 66)	22 (16 to 29)	91 (84 to 95)	1.63 (1.14 to 2.33)	0.53 (0.26 to 1.05)
mRAI (≥8p)	0.76 (0.65 to 0.86)	<0.001	75 (51 to 91)	65 (55 to 74)	27 (21 to 35)	94 (87 to 97)	2.14 (1.5 to 3)	0.39 (0.18 to 0.83)
**Basal biomarker performance**								
HSP-72 (>0.2 ng/ml)	0.56 (0.41 to 0.71)	0.39	39 (17 to 64)	73 (63 to 81)	29 (11 to 32)	87 (82 to 91)	1.44 (0.75 to 2.79)	0.84 (0.57 to 1.23)
NGAL (>152 ng/ml)	0.62 (0.48 to 0.76)	0.10	35 (14 to 61)	77 (67 to 84)	20 (11 to 35)	87 (83 to 91)	1.53 (0.73 to 3.21)	0.84 (0.58 to 1.21)
TIMP-2×IGFBP7 ((ng/mL)^2^/1000)	0.76 (0.63 to 0.89)	0.001	64 (38 to 85)	83 (74 to 90)	39 (27 to 53)	93 (88 to 96)	3.88 (2.22 to 6.78)	0.42 (0.22 to 0.81)
CCL14 (>250 pg/ml)	0.57 (0.43 to 0.70)	0.34	70 (44 to 89)	58 (48 to 68)	22 (16 to 29)	92 (84 to 96)	1.7 (1.16 to 2.49)	0.5 (0.24 to 1.07)
KIM-1 (>1.4 ng/ml)	0.56 (0.44 to 0.69)	0.35	55 (30 to 78)	53 (42 to 63)	18 (12 to 26)	86 (78 to 91)	1.19 (0.74 to 1.89)	0.84 (0.48 to 1.45)

Predictive performance and discrimination *were* determined for the primary outcome: severe AKI within day 1–3 by Kidney Disease Improving Global Outcomes stages 2–3 change of creatinine from baseline. Area under the receiver operating characteristic curve-receiver-operating characteristic was performed on renal angina indices and urinary biomarkers on day 0 of admission. Cutoff values used for each biomarker were determined based on Youden index on individual urinary biomarkers for the population tested, urinary biomarkers included: heat shock protein 72 *n*=122; heat shock protein 72 *n*=117; tissue inhibitor of metalloproteinase-2×IGF-binding protein 7 *n*=119; C–C motif chemokine ligand 14 *n*=118; kidney injury molecule-1 *n*=112 patients. Results are expressed with 95% confidence interval. AUC, area under the receiver operating characteristic curve; CCL14, C–C motif chemokine ligand 14; CI, confidence interval; HSP-72, heat shock protein 72; IGFBP7, IGF-binding protein 7; KIM-1, kidney injury molecule-1; LR, likelihood ratio; mRAI, modified renal angina index; NGAL, neutrophil gelatinase-associated lipocalin; NPV, negative predictive value; PPV, positive predictive value; RAI, renal angina index; TIMP-2, tissue inhibitor of metalloproteinase-2.

### Urinary Biomarkers and Prediction of Severe AKI

Urinary biomarkers were measured in 118 patients (88% of the cohort) due to missing urine samples after patient inclusion, primarily related to challenges in sample recollection or processing errors. Among the urinary biomarkers tested only [TIMP‐2]×[IGFBP7] independently predicted severe AKI, with moderate performance (AUC, 0.76; 95% CI, 0.63 to 0.89). Using a previously reported cutoff value (2 ng/ml^2^ per 1000), this urinary biomarker demonstrated high specificity and NPV. No other urinary biomarker outperformed the RAIs in predicting the primary outcome (Figure 2, D–H and Table 3).

### Combined Use of RAIs and Urinary Biomarkers

Decision trees were constructed for each RAIs and urinary biomarkers combination (Supplemental Figure 1), stratifying patients based on predefined decision thresholds. The probability of severe AKI was estimated as the proportion of severe AKI cases within each RAI-biomarker subgroup (Supplemental Table 3). Combining urinary biomarkers with RAIs improved the predictive performance for severe AKI in all models, with statistically significant improvement only observed when [TIMP‐2]×[IGFBP7] was added: Matsuura RAI (AUC 0.77, *P* = 0.01), and Del-Toro-Cisneros RAI (AUC 0.80, *P* = 0.04). For mRAI, combining it with [TIMP‐2]×[IGFBP7] yielded the highest AUC in our analyses (0.82; 95% CI 0.72 to 0.93); however, compared with mRAI alone, this improvement did not reach statistical significance (Supplemental Table 4).

Sequential assessment using decision trees enhanced risk stratification and reclassification among RAI+ patients. This approach increased predictive accuracy for severe AKI regardless of the RAI-biomarker pairing (Figure 2, I–W and Table 4). The NRI gains were due to correct reclassification of non-events. Significant NRI values were found for [TIMP‐2]×[IGFBP7] across all RAIs, whereas C–C motif chemokine ligand 14 was significant only with Matsuura RAI (NRI −0.64, *P* = 0.014; Supplemental Table 4).

**Table 4:** Diagnostic performance of renal angina indices in combination with urinary biomarkers

Index/Biomarker Combination	AUC (95% CI)	*P* Value	Sensitivity (%) (95% CI)	Specificity (%) (95% CI)	PPV (%) (95% CI)	NPV (%) (95% CI)	LR+ (95% CI)	LR− (95% CI)	Accuracy (95% CI)
**RAI Matsuura (≥10p)**	0.63 (0.48 to 0.78)	0.05	60 (36 to 80)	61 (52 to 70)	15 (9 to 22)	89 (83 to 93)	1.55 (1 to 2.38)	0.65 (0.37 to 1.14)	61 (52 to 69)
HSP-72	0.63 (0.47 to 0.79)	0.07	22 (6 to 48)	90 (83 to 95)	29 (12 to 53)	87 (84 to 90)	2.31 (0.81 to 6.58)	0.86 (0.67 to 1.11)	80 (72 to 87)
NGAL	0.65 (0.51 to 0.80)	0.04	24 (7 to 50)	89 (81 to 94)	27 (12 to 50)	87 (84 to 90)	2.14 (0.77 to 5.95)	0.86 (0.65 to 1.13)	78 (71 to 86)
TIMP-2×IGFBP7	0.77 (0.63 to 0.91)	<0.001	35 (14 to 67)	96 (90 to 99)	90 (86 to 93)	60 (32 to 83)	8.91 (2.8 to 28.32)	0.67 (0.47 to 0.96)	87 (80 to 93)
CCL14	0.57 (0.41 to 0.74)	0.3	41 (18 to 67)	80 (71 to 87)	26 (15 to 41)	89 (84 to 92)	2.08 (1.04 to 4.15)	0.73 (0.49 to 1.1)	75 (66 to 82)
KIM-1	0.59 (0.43 to 0.76)	0.19	33 (13 to 59)	83 (74 to 90)	27 (15 to 45)	87 (82 to 90)	1.96 (0.89 to 4.32)	0.8 (0.57 to 1.13)	75 (66 to 83)
**RAI Del-Toro-Cisneros (≥8p)**	0.71 (0.60 to 0.82)	0.002	70 (45 to 88)	57 (47 to 66)	22 (16 to 29)	91 (84 to 95)	1.63 (1.14 to 2.33)	0.53 (0.26 to 1.05)	59 (50 to 67)
HSP-72	0.69 (0.57 to 0.80)	0.01	22 (6 to 48)	88 (80 to 94)	25 (10 to 48)	87 (83 to 89)	1.91 (0.69 to 5.26)	0.88 (0.68 to 1.14)	79 (70 to 85)
NGAL	0.68 (0.56 to 0.80)	0.02	24 (7 to 50)	88 (80 to 94)	25 (9 to 22)	87 (84 to 90)	1.96 (0.72 to 5.37)	0.87 (0.66 to 1.14)	79 (70 to 86)
TIMP-2×IGFBP7	0.80 (0.68 to 0.92)	<0.001	47 (23 to 72)	91 (84 to 96)	47 (29 to 66)	91 (87 to 94)	5.33 (2.39 to 11.89)	0.58 (0.37 to 0.91)	85 (88 to 90)
CCL14	0.63 (0.49 to 0.77)	0.07	47 (23 to 72)	88 (79 to 94)	42 (26 to 61)	90 (95 to 93)	3.85 (1.82 to 8.14)	0.6 (0.38 to 0.95)	74 (65 to 81)
KIM-1	0.67 (0.54 to 0.79)	0.02	39 (17 to 64)	81 (71 to 88)	28 (16 to 44)	87 (83 to 91)	2.03 (1 to 4.14)	0.76 (0.52 to 1.11)	75 (65 to 82)
**mRAI (≥8p)**	0.76 (0.68 to 0.83)	<0.001	75 (51 to 91)	65 (55 to 74)	27 (21 to 35)	94 (87 to 97)	2.14 (1.5 to 3)	0.39 (0.18 to 0.83)	66 (58 to 74)
HSP-72	0.72 (0.60 to 0.84)	<0.001	28 (10 to 53)	93 (86 to 97)	42 (20 to 67)	88 (85 to 91)	4.13 (1.47 to 11.6)	0.77 (0.56 to 1.04)	83 (76 to 90)
NGAL	0.70 (0.57 to 0.83)	0.007	30 (10 to 56)	91 (84 to 96)	36 (17 to 59)	88 (84 to 91)	3.27 (1.25 to 8.6)	0.78 (0.57 to 1.06)	82 (74 to 88)
TIMP-2×IGFBP7	0.82 (0.72 to 0.93)	<0.001	41 (18 to 67)	95 (89 to 98)	58 (33 to 80)	91 (87 to 93)	8.4 (3 to 23)	0.62 (0.41 to 0.92)	87 (80 to 93)
CCL14	0.71 (0.60 to 0.82)	0.005	53 (28 to 77)	82 (73 to 89)	33 (21 to 48)	91 (86 to 94)	2.97 (1.61 to 5.5)	0.57 (0.34 to 0.96)	78 (69 to 85)
KIM-1	0.68 (0.54 to 0.82)	0.01	39 (17 to 64)	82 (73 to 89)	29 (17 to 46)	87 (83 to 91)	2.15 (1.05 to 4.4)	0.75 (0.51 to 1.09)	75 (66 to 83)

AUC, area under the receiver operating characteristic curve; CCL14, C–C motif chemokine ligand 14; CI, confidence interval; HSP-72, heat shock protein 72; IGFBP7, IGF-binding protein 7; KIM-1, kidney injury molecule-1; LR, likelihood ratio; mRAI, modified renal angina index; NGAL, neutrophil gelatinase-associated lipocalin; NPV, negative predictive value; PPV, positive predictive value; RAI, renal angina index; TIMP-2, tissue inhibitor of metalloproteinase-2.

Sensitivity analyses using multiple imputation produced results consistent with the complete-case analysis. AUC estimates did not differ significantly, indicating that missing biomarker data had minimal impact on model performance (Supplemental Table 5).

## Discussion

In this prospective study, we validated the hypothesis that incorporating urinary biomarkers into clinical risk stratification models, specifically RAIs, enhances the prediction of clinically significant severe AKI in critically ill adult patients. Using three different RAIs in combination with five urinary biomarkers, we found that locally derived mRAI, combined with [TIMP-2]×[IGFBP7] provided the highest absolute discrimination for severe AKI within 72 hours of ICU admission. Although the combined mRAI+[TIMP-2]×[IGFBP7] model achieved the numerically highest AUC (AUC, 0.82; 95% CI, 0.72 to 0.93), the difference compared with mRAI alone was not statistically significant. This limitation underscores the need for replication in larger, external cohorts before firm conclusions can be drawn.

Although our observed severe AKI incidence (15%) is lower than in some previous RAI/biomarker studies,^8,15,21^ this is likely explained by strict exclusion criteria (*e.g*., CKD, prevalent AKI) and the 72-hour outcome window, which specifically targeted early AKI prediction. Similar incidence rates have been reported in other prospective ICU studies^6,22^ with comparable design.

Although multiple biomarkers have been proposed for early detection of AKI, their diagnostic performance is often suboptimal when applied outside an appropriate clinical framework. The RAI is designed to identify patients with early or incipient AKI who are at high risk for progression to severe AKI, rather than detecting AKI in its entirely preclinical phase. In this context, the use of RAIs enabled early identification of patients at high risk for developing severe AKI, and when used to guide biomarker testing, significantly improved the diagnostic accuracy, particularly [TIMP-2]×[IGFBP7]. This sequential approach optimizes early risk stratification and facilitates timely intervention.

The RAI, originally validated in pediatric populations, showed excellent predictive performance.^7^ In adult ICU cohorts, adaptations have been necessary to recalibrate clinical risk variables.^23^ Matsuura *et al.*^6^ evaluated the RAI in an Asian adult ICU population and found modest performance (AUC, 0.63; 95% CI, 0.53 to 0.73), a finding mirrored in our cohort (AUC, 0.63; 95% CI, 0.48 to 0.78) using the original cutoff score of 10. Despite incorporating CKD as a component, performance was unchanged in our study after excluding patients with moderate CKD. Conversely, Del-Toro-Cisneros RAI^8^ was developed in a more homogeneous population with coronavirus disease 2019–related respiratory failure, which may limit its applicability to broader ICU populations. In our cohort, the Del-Toro-Cisneros RAI achieved fair performance (AUC, 0.71; 95% CI, 0.60 to 0.82), albeit with a higher NPV (60% versus 91%), likely attributable to differences in AKI incidence. These findings suggest that the context and population in which a RAI is derived strongly influence its predictive capacity, and support the need for locally adapted models such as the mRAI.

Additional RAIs tailored for adult ICU settings, such as the Ortiz-Soriano *et al.*^24^ model, integrate static risk factors with early changes in SCr and fluid balance, achieving good discrimination (AUC, 0.76; 95% CI, 0.74 to 0.79). However, reliance on 24-hour serial measurements delays risk stratification and limits early intervention, particularly given demographic differences compared to our cohort.

Using logistic regression, we derived mRAI by weighting candidate predictors. The mRAI demonstrated superior performance (AUC, 0.76; 95% CI, 0.65 to 0.86), with improved sensitivity and NPV. The effects of vasopressor use and MV were largely subsumed by SOFA score, enabling a parsimonious model. Limiting baseline comorbidities to cirrhosis may have attenuated sensitivity and reduced generalizability. Inclusion of lactate, although not traditionally part of RAI models, is biologically plausible, given its association with AKI development and adverse outcomes.^25–28^

While RAIs exhibit high NPV, false‐positive remains meaningful. Whereas urinary biomarkers alone are not superior to RAIs, their sequential integration following risk stratification enhances diagnostic precision, independent of baseline RAI performance. As with troponin, most informative in patients with high pretest probability and often prompting invasive intervention, AKI biomarkers are most valuable in high‐risk settings, where they enable risk stratification and tailored supportive care, rather than guiding a single definitive treatment.

Previous studies support this concept. Menon *et al.*^21^ demonstrated that neutrophil gelatinase-associated lipocalin integration markedly improved AKI prediction (AUC, 0.97; 95% CI, 0.93 to 1.00) but maintained low positive predictive value (PPV). Matsuura *et al.*^6^ and Goldstein *et al.*^15^ showed that urinary biomarkers improved PPV among RAI-positive patients, although early misclassification remained a limitation. Basu *et al.*^16^ further confirmed that urinary biomarkers enhanced model discrimination by correctly reclassifying patients without events into lower-risk categories. In alignment with these findings, most of the observed reclassification gains were due to correct reclassification of patients who did not develop AKI, meaning that the addition of biomarkers improved identification of low-risk individuals. This has relevant clinical implications, as recognizing patients at low risk could reduce unnecessary diagnostic testing, repeated biomarker measurements, and unwarranted interventions, thereby promoting a more efficient allocation of resources in the ICU. Using a [TIMP-2]×[IGFBP7] cutoff of 2 (ng/ml)^2^/1000, positivity among RAI(+) patients markedly increased PPV and facilitated risk downgrading among biomarker-negative individuals, thus improving AUCs (RAI Matsuura AUC, 0.77; RAI Del-Toro-Cisneros AUC, 0.8; mRAI AUC, 0.83). Consistent with our findings, Jia *et al.*^29^ reported superior predictive value of [TIMP-2]×[IGFBP7] in high-risk ICU patients, particularly when clinical trajectories are uncertain.

In this context, RAI discrimination is pivotal for initial screening. Biomarkers then function as second-tier tests—refining risk chiefly in RAI-positive patients when baseline models are strong (*e.g*., mRAI) and assuming a larger role when discrimination is weaker (*e.g*., Matsuura) by reclassifying both RAI-positive and RAI-negative patients, underscoring their incremental value.

The heterogeneity of ICU admission diagnoses in our cohort was intentional, aiming to reflect real-world case mixes and enhance the generalizability of our findings. Nevertheless, we recognize that predictive performance may vary in specific subgroups (*e.g*., septic shock, post-cardiac surgery), and dedicated studies in these populations will be necessary to determine whether biomarker-enhanced RAIs provide additional discriminatory value in more homogeneous settings. Generalizability is further limited by our single-center design and the fact that nearly all participants were of Mexican ethnicity.

Among the five urinary biomarkers, [TIMP-2]×[IGFBP7] was the most informative adjunct across RAIs; decision-tree analyses and net reclassification improvement confirmed its incremental predictive value. Pairing simplified RAIs with urinary biomarkers offers a pragmatic strategy for early AKI risk stratification, enhancing early identification, guiding timely interventions, and potentially improving outcomes in critically ill patients.

Although no specific pharmacologic therapy can reverse AKI, early risk identification allows timely implementation of nephroprotective measures: optimizing hemodynamics, guiding fluid management, discontinuing nephrotoxins, adjusting drug dosing, and prioritizing nephrology consultation. Early detection also facilitates enrollment in interventional trials and supports shared decision‐making regarding KRT planning.

Although advanced predictive tools such as deep learning and novel imaging are emerging,^30,31^ their ICU use remains limited by cost and infrastructure, particularly in low- and middle-income countries. By contrast, sequential RAI-urinary biomarker strategies are simple, reproducible, and feasible at the bedside, especially with US Food and Drug Administration–cleared assays such as NephroCheck^32^ for [TIMP-2]×[IGFBP7]. Recent Acute Disease Quality Initiative^5^ recommendations endorse biomarker-guided AKI risk stratification, underscoring its translational potential and the need for multicenter validation in diverse populations.

This study has several limitations. First, the relatively small sample size, and in particular the limited number of patients who developed severe AKI (*n*=20), is a major limitation of this study. This constraint reduces statistical power, especially for subgroup analyses, and therefore our findings should be considered hypothesis-generating. Larger, multicenter studies will be necessary to externally validate these results and confirm their generalizability. Second, urine output criteria, a sensitive marker for AKI diagnosis, were not included due to incomplete and heterogeneous documentation across ICUs, potentially leading to underestimation of AKI incidence. Third, biomarker measurements were unavailable in 12% of patients. However, sensitivity analysis using multiple imputation indicated that missingness was unlikely to bias results, as model performance was consistent across imputed and complete-case datasets. Four, the study focused exclusively on short-term AKI diagnosis within 72 hours and did not explore long-term outcomes such as renal recovery, persistent AKI, or mortality. Future investigations should incorporate these end points to fully elucidate the utility of sequential risk stratification in critically ill populations.

In conclusion, sequential risk stratification in critically ill patients using RAIs followed by targeted urinary biomarkers profiling significantly enhanced early prediction of severe AKI. Clinical RAIs models alone demonstrated poor to fair predictive performance for severe AKI within 72 hours of ICU admission; however, integration with urinary biomarkers, particularly mRAI combined with [TIMP-2]×[IGFBP7], yielded higher absolute performance. These findings suggest that multimodal diagnostic strategies may improve early AKI detection and guide patient-centered critical care management; however, they should be regarded as hypothesis-generating and require replication in larger, external cohorts before clinical implementation.

## Supplementary Material

## Data Availability

Original data generated for the study will be made available upon reasonable request to the corresponding author. Data Type: Observational Data. Reason for Restricted Access: The informed-consent documents and Research Ethics Committee approvals did not authorize public posting of individual-level data. Given the small cohort size and the presence of potentially identifying clinical combinations, the risk of re-identification is non-trivial under institutional policy and Mexican privacy law. Deidentified data (clinical variables and biomarker summary values), a data dictionary, and analysis code will be provided upon reasonable request to the corresponding author for noncommercial academic use under a Data Use Agreement and, when applicable, documentation of local Institutional Review Board (IRB) and a Research Ethics Committee (REC) approval or exemption.

## Declarative Statements

This study includes clinical experimentation and received Institutional Review Board or Ethics Committee approval. All patients provided written informed consent. This study includes clinical experimentation and complies with the Declaration of Helsinki.

## Supplemental Material

Supplemental Table 1. Logistic regression analysis for development of mRAI.

Supplemental Table 2. Derivation of the mRAI.

Supplemental Table 3. Probability of AKI per subpopulation of RAI and biomarker combination.

Supplemental Table 4. Predictive performance and discrimination for severe AKI within 72 hours by the RAI in combination with urinary biomarkers.

Supplemental Table 5. Sensitivity analysis of model performance after multiple imputation by predictive mean matching compared with complete-case analysis.

Supplemental Figure 1. AKI probability after incorporating biomarkers into the RAI model into decision-tree.
