# Supplementary material for: Renal Angina Indices and Urinary Biomarkers: A Combined Approach to Predict Acute Kidney Injury in Critically Ill Patients
Source: Kidney360. 2025 Nov 12;7(1):61–71. doi: 10.34067/KID.0000001020 (PMC12890014; doi:10.34067/KID.0000001020)
Supplement: Supplementary file 1 [file kidney360-7-061-s001.pdf]

ASN American Society of Nephrology | JASN Journal of the American Society of Nephrology | KIDNEY360 Accessing Our World From Every Angle | CJASN Clinical Journal of the American Society of Nephrology

ASN Journal Disclosure Form

As per ASN journal policy, I have disclosed any financial relationships or commitments I have held in the past 36 months as included below. I have listed my Current Employer below to indicate there is a relationship requiring disclosure. If no relationship exists, my Current Employer is not listed.

N. Del Toro-Cisneros reports the following:
Employer: INSTITUTO NACIONAL DE CIENCIAS M?DICAS Y NUTRICI?N SALVADOR ZUBIR?N; Consultancy: Vantive; and Speakers Bureau: Vantive.

I understand that the information above will be published within the journal article, if accepted, and that failure to comply and/or to accurately and completely report the potential financial conflicts of interest could lead to the following: 1) Prior to publication, article rejection, or 2) Post-publication, sanctions ranging from, but not limited to, issuing a correction, reporting the inaccurate information to the authors' institution, banning authors from submitting work to ASN journals for varying lengths of time, and/or retraction of the published work.

Name: Noemi Del Toro-Cisneros
Manuscript ID: K360-2025-000617R1
Manuscript Title: Renal Angina Indices and Urinary Biomarkers: A Combined Approach to Predict Acute Kidney Injury in Critically Ill Patients
Date of Completion: September 8, 2025
Disclosure Updated Date: May 21, 2025

ASN American Society of Nephrology | JASN Journal of the American Society of Nephrology | KIDNEY360 Accessing Our World From Every Angle | CJASN Clinical Journal of the American Society of Nephrology

ASN Journal Disclosure Form

As per ASN journal policy, I have disclosed any financial relationships or commitments I have held in the past 36 months as included below. I have listed my Current Employer below to indicate there is a relationship requiring disclosure. If no relationship exists, my Current Employer is not listed.

P. Galindo reports the following:
Employer: Hospital General de México Dr. Eduardo Liceaga; and Speakers Bureau: BAXTER/ VANTIVE.

I understand that the information above will be published within the journal article, if accepted, and that failure to comply and/or to accurately and completely report the potential financial conflicts of interest could lead to the following: 1) Prior to publication, article rejection, or 2) Post-publication, sanctions ranging from, but not limited to, issuing a correction, reporting the inaccurate information to the authors' institution, banning authors from submitting work to ASN journals for varying lengths of time, and/or retraction of the published work.

Name: Pablo E. Galindo
Manuscript ID: K360-2025-000617R1
Manuscript Title: Renal Angina Indices and Urinary Biomarkers: A Combined Approach to Predict Acute Kidney Injury in Critically Ill Patients
Date of Completion: September 10, 2025
Disclosure Updated Date: September 10, 2025

ASN American Society of Nephrology | JASN Journal of the American Society of Nephrology | KIDNEY360 Accessing Our World From Every Angle | CJASN Clinical Journal of the American Society of Nephrology

ASN Journal Disclosure Form

As per ASN journal policy, I have disclosed any financial relationships or commitments I have held in the past 36 months as included below. I have listed my Current Employer below to indicate there is a relationship requiring disclosure. If no relationship exists, my Current Employer is not listed.

K. Linares has nothing to disclose.

I understand that the information above will be published within the journal article, if accepted, and that failure to comply and/or to accurately and completely report the potential financial conflicts of interest could lead to the following: 1) Prior to publication, article rejection, or 2) Post-publication, sanctions ranging from, but not limited to, issuing a correction, reporting the inaccurate information to the authors' institution, banning authors from submitting work to ASN journals for varying lengths of time, and/or retraction of the published work.

Name: Kalra Linares
Manuscript ID: K360-2025-000617R
Manuscript Title: Renal Angina Indices and Urinary Biomarkers: A Combined Approach to Predict Acute Kidney Injury in Critically Ill Patients,
Date of Completion: September 11, 2025
Disclosure Updated Date: September 11, 2025

ASN American Society of Nephrology | JASN Journal of the American Society of Nephrology | KIDNEY360 Accessing Our World From Every Angle | CJASN Clinical Journal of the American Society of Nephrology

ASN Journal Disclosure Form

As per ASN journal policy, I have disclosed any financial relationships or commitments I have held in the past 36 months as included below. I have listed my Current Employer below to indicate there is a relationship requiring disclosure. If no relationship exists, my Current Employer is not listed.

M. López Ruelas reports the following:
Employer: Instituto Nacional de Ciencias Médicas y Nutrición "Salvador Zubirán"

I understand that the information above will be published within the journal article, if accepted, and that failure to comply and/or to accurately and completely report the potential financial conflicts of interest could lead to the following: 1) Prior to publication, article rejection, or 2) Post-publication, sanctions ranging from, but not limited to, issuing a correction, reporting the inaccurate information to the authors' institution, banning authors from submitting work to ASN journals for varying lengths of time, and/or retraction of the published work.

Name: María José M López Ruelas
Manuscript ID: K360-2025-000617R1
Manuscript Title: "Renal Angina Indices and Urinary Biomarkers: A Combined Approach to Predict Acute Kidney Injury in Critically Ill Patients"
Date of Completion: September 8, 2025
Disclosure Updated Date: September 8, 2025

ASN
American Society of Nephrology
JASN
Journal of the American Society of Nephrology
KIDNEY360
Accessing Our World From Every Angle
CJASN
Clinical Journal of the American Society of Nephrology

ASN Journal Disclosure Form

As per ASN journal policy, I have disclosed any financial relationships or commitments I have held in the past 36 months as included below. I have listed my Current Employer below to indicate there is a relationship requiring disclosure. If no relationship exists, my Current Employer is not listed.

Y. Mercado Hernández has nothing to disclose.

I understand that the information above will be published within the journal article, if accepted, and that failure to comply and/or to accurately and completely report the potential financial conflicts of interest could lead to the following: 1) Prior to publication, article rejection, or 2) Post-publication, sanctions ranging from, but not limited to, issuing a correction, reporting the inaccurate information to the authors' institution, banning authors from submitting work to ASN journals for varying lengths of time, and/or retraction of the published work.

Name: Yazmin Alejandra Mercado Hernández
Manuscript ID: K360-2025-000617R1
Manuscript Title: Renal Angina Indices and Urinary Biomarkers: A Combined Approach to Predict Acute Kidney Injury in Critically Ill Patients
Date of Completion: September 9, 2025
Disclosure Updated Date: September 9, 2025

ASN
American Society of Nephrology
JASN
Journal of the American Society of Nephrology
KIDNEY360
Accessing Our World From Every Angle
CJASN
Clinical Journal of the American Society of Nephrology

ASN Journal Disclosure Form

As per ASN journal policy, I have disclosed any financial relationships or commitments I have held in the past 36 months as included below. I have listed my Current Employer below to indicate there is a relationship requiring disclosure. If no relationship exists, my Current Employer is not listed.

R. Ortega has nothing to disclose.

I understand that the information above will be published within the journal article, if accepted, and that failure to comply and/or to accurately and completely report the potential financial conflicts of interest could lead to the following: 1) Prior to publication, article rejection, or 2) Post-publication, sanctions ranging from, but not limited to, issuing a correction, reporting the inaccurate information to the authors' institution, banning authors from submitting work to ASN journals for varying lengths of time, and/or retraction of the published work.

Name: Rosario Guadalupe Hernandez Ortega
Manuscript ID: K360-2025-000617R1
Manuscript Title: Renal Angina Indices and Urinary Biomarkers: A Combined Approach to Predict Acute Kidney Injury in Critically Ill Patients
Date of Completion: September 9, 2025
Disclosure Updated Date: September 9, 2025

ASN American Society of Nephrology
JASN Journal of the American Society of Nephrology
KIDNEY360 Accessing Our World From Every Angle
CJASN Clinical Journal of the American Society of Nephrology

ASN Journal Disclosure Form

As per ASN journal policy, I have disclosed any financial relationships or commitments I have held in the past 36 months as included below. I have listed my Current Employer below to indicate there is a relationship requiring disclosure. If no relationship exists, my Current Employer is not listed.

C. Cruz Rivera has nothing to disclose.

I understand that the information above will be published within the journal article, if accepted, and that failure to comply and/or to accurately and completely report the potential financial conflicts of interest could lead to the following: 1) Prior to publication, article rejection, or 2) Post-publication, sanctions ranging from, but not limited to, issuing a correction, reporting the inaccurate information to the authors' institution, banning authors from submitting work to ASN journals for varying lengths of time, and/or retraction of the published work.

Name: Cristino Cruz Rivera
Manuscript ID: K360-2025-000617R1
Manuscript Title: Renal Angina Indices and Urinary Biomarkers: A Combined Approach to Predict Acute Kidney Injury in Critically Ill Patients
Date of Completion: October 15, 2025
Disclosure Updated Date: October 9, 2025

ASN American Society of Nephrology
JASN Journal of the American Society of Nephrology
KIDNEY360 Accessing Our World From Every Angle
CJASN Clinical Journal of the American Society of Nephrology

ASN Journal Disclosure Form

As per ASN journal policy, I have disclosed any financial relationships or commitments I have held in the past 36 months as included below. I have listed my Current Employer below to indicate there is a relationship requiring disclosure. If no relationship exists, my Current Employer is not listed.

O. Vega reports the following:
Employer: National Institute of Medical Sciences; Consultancy: Baxter; Research Funding: Baxter and Fresenius; Advisory or Leadership Role: AstraZeneca;; and Speakers Bureau: Amgen; Baxter; Boehringer-Lilly, AstraZeneca.

I understand that the information above will be published within the journal article, if accepted, and that failure to comply and/or to accurately and completely report the potential financial conflicts of interest could lead to the following: 1) Prior to publication, article rejection, or 2) Post-publication, sanctions ranging from, but not limited to, issuing a correction, reporting the inaccurate information to the authors' institution, banning authors from submitting work to ASN journals for varying lengths of time, and/or retraction of the published work.

Name: Olynka Vega
Manuscript ID: K360-2025-000617R1
Manuscript Title: Renal Angina Indices and Urinary Biomarkers: A Combined Approach to Predict Acute Kidney Injury in Critically Ill Patients
Date of Completion: September 10, 2025
Disclosure Updated Date: September 10, 2025

ASN American Society of Nephrology
JASN Journal of the American Society of Nephrology
KIDNEY360 Accessing Our World From Every Angle
CJASN Clinical Journal of the American Society of Nephrology

ASN Journal Disclosure Form

As per ASN journal policy, I have disclosed any financial relationships or commitments I have held in the past 36 months as included below. I have listed my Current Employer below to indicate there is a relationship requiring disclosure. If no relationship exists, my Current Employer is not listed.

J. Villegas-Gamas reports the following:
Employer: Instituto Nacional de Ciencias Médicas y Nutrición Salvador Zubirán; IGSA MEDICAL; Ownership Interest: TAKE-TWO INTERACTIVE SOFTWARE INC.; GAMESTOP CORP.; Honoraria: Laboratorio SILANES.; and Speakers Bureau: Laboratorio SILANES.

I understand that the information above will be published within the journal article, if accepted, and that failure to comply and/or to accurately and completely report the potential financial conflicts of interest could lead to the following: 1) Prior to publication, article rejection, or 2) Post-publication, sanctions ranging from, but not limited to, issuing a correction, reporting the inaccurate information to the authors' institution, banning authors from submitting work to ASN journals for varying lengths of time, and/or retraction of the published work.

Name: Juan M Villegas-Gamas
Manuscript ID: K360-2025-000617R1
Manuscript Title: Renal Angina Indices and Urinary Biomarkers: A Combined Approach to Predict Acute Kidney Injury in Critically Ill Patients
Date of Completion: September 10, 2025
Disclosure Updated Date: September 10, 2025

ASN American Society of Nephrology | JASN Journal of the American Society of Nephrology | KIDNEY360 Accessing Our World From Every Angle | CJASN Clinical Journal of the American Society of Nephrology

ASN Journal Disclosure Form

As per ASN journal policy, I have disclosed any financial relationships or commitments I have held in the past 36 months as included below. I have listed my Current Employer below to indicate there is a relationship requiring disclosure. If no relationship exists, my Current Employer is not listed.

E. Zuñiga Gonzalez reports the following:
Employer: Instituo Nacional de Ciencias Medicas de Nutrición

I understand that the information above will be published within the journal article, if accepted, and that failure to comply and/or to accurately and completely report the potential financial conflicts of interest could lead to the following: 1) Prior to publication, article rejection, or 2) Post-publication, sanctions ranging from, but not limited to, issuing a correction, reporting the inaccurate information to the authors' institution, banning authors from submitting work to ASN journals for varying lengths of time, and/or retraction of the published work.

Name: Erick Yasar Zuñiga Gonzalez
Manuscript ID: K360-2025-000617R1
Manuscript Title: Renal Angina Indices and Urinary Biomarkers: A Combined Approach to Predict Acute Kidney Injury in Critically Ill Patients
Date of Completion: September 8, 2025
Disclosure Updated Date: September 8, 2025
