# Supplementary material for: Renal Angina Indices and Urinary Biomarkers: A Combined Approach to Predict Acute Kidney Injury in Critically Ill Patients
Source: Kidney360. 2025 Nov 12;7(1):61–71. doi: 10.34067/KID.0000001020 (PMC12890014; doi:10.34067/KID.0000001020)
Supplement: Supplementary file 2 [file kidney360-7-061-s002.pdf]

Supplemental Materials Table of Contents

Supplemental Table 1. Logistic regression analysis for development of modified renal angina index

Supplemental Table 2. Derivation of the modified renal angina index.

Supplemental Table 3. Probability of AKI per subpopulation of RAI and biomarker combination.

Supplemental Table 4. Predictive performance and discrimination for severe AKI by the RAI in combination with urinary biomarkers.

Supplemental Table 5. Sensitivity analysis of model performance after multiple imputation by predictive mean matching (PMM) compared with complete-case analysis.

Supplemental Figure 1. AKI probability after incorporating biomarkers into the Renal Angina Index Model into decision tree.

Supplemental Table 1. Logistic regression analysis for development of modified renal angina index

Logistic regression analysis for acute kidney injury (KDIGO2-3) at Day3; univariate and multivariate.
Model 1: Variables with statistical significance (p<0.1) were included in a multivariate logistic regression model. The best model was constructed using forward stepwise selection, with an entry criterion of p<0.1 and a removal criterion of p>0.3.
Model 2: Model adjusted for age and sex.

	UNIVARIATE		MULTIVARIATE			
			MODEL 1	p-value	MODEL 2	p-value
	OR (95% CI)	p-value	ORa (95% CI)		ORa (95% CI)	
**Age, years**	1.00 (0.98-1.032)	0.43				
**Female vs male**	0.70 (0.26-1.92)	0.49				
**Sepsis**	1.59 (0.83-6)	0.11				
**CNS**	0.36 (0.08-1.68)	0.19				
**Circulatory dysfunction**	0.35 (0.076-1.60)	0.17				
**Post-operative**	1.51 (0.49-4.6)	0.47				
**Liver failure**	3.14 (0.27-36.4)	0.36				
BMI ≥30 Kg/m²	1.99 (0.71-5.59)	0.18				
Non-renal SOFA ≥6	3.77 (1.27-11.17)	**0.01**	3.23 (0.84-12.39)	0.087	3.35 (0.94-11.88)	0.06
**Charlson index, por point**	1.13 (0.92-1.39)	0.21				
**Diabetes**	1.369 (0.47-3.9)	0.56				
**Cirrhosis**	5.43 (1.66-17.7)	**0.005**	4.16 (1-17.3)	**0.05**	3.77 (0.93-15.3)	**0.063**
**Heart disease**	0.74 (0.26-2.1)	0.57				
**Rheumatologic**	0.91 (0.52-6.07)	0.35				
**Malignancy**	1.77 (0.44-7.05)	0.41				
**CKD**	3.26 (0.55-19.21)	0.19				
**Pulmonary disease**	0.58 (0.07-4.83)	0.61				
**Ventilatory support**	1.35 (0.56-3.6)	0.54				
**Vasopressor support**	2.24 (0.69-7.1)	0.17				
**>1 vasopressor**	5.70 (1.94-6.773)	**0.002**				
**Lactate >2 mmol/L**	4.75 (1.63-13.89)	**0.004**	7.29 (2.04-26.02)	**0.002**	6 (1.86-19.5)	**0.003**
**Hemoglobin >10 g/dl**	0.65 (0.24-1.75)	0.39				
**Albumin >3 g/dl**	0.62 (0.22-1.79)	0.38				
**Total bilirubin >2mg/dl**	5.03 (1.78-14.2)	**0.002**				
**Sodium mEq/L**	0.97 (0.89-1.059)	0.50				
**Potassium mEq/L**	0.59 (0.59-2.68)	0.55				
**Bicarbonate <16 mmol/L**	0.32 (0.54-1.92)	0.21				
**ALT >28 U/L**	2.76 (1.013-7.6)	**0.04**				

OR, odds ratio, ORa, adjusted odds ratio; CI, confidence interval; CNS, Central nervous system; BMI, body mass index, SOFA, Sequential Organ Failure Assessment; CKD, chronic kidney disease

Supplemental Table 2. Derivation of the modified renal angina index

We developed a modified renal angina index, under patient's risk of AKI in this population. The condition component was determined based on logistic regression analysis, and those significantly associated with significant AKI in the multivariable logistic regression analysis were assigned risk scores according to the odds ratio. Conditions selected were SOFA score ≥6, cirrhosis and lactate ≥2mmol/l. RAI was calculated by multiplicative index based on change of serum creatinine and the sum of risk scores of selected patient conditions.
The composite range of the RAI is therefore: 1, 2, 3, 4, 6, 8, 9, 12, 18, 24, 36, 40, 48, 72 and 96.

The table depicts the RAI cut-off scores in increasing order and associated predictive performance and Youden's index (J-statistic). Based on the most optimal Youden's index and highest negative predictive value (ruling out the likelihood of subsequent AKI), a RAI > 8 was indicative of fulfilling renal angina.

	#	# Day1-3 AKI	Sens	Spec	PPV	NPV	LR+	LR-	J-statistic
**≥1**	134	20	100	0	15	0	1	0	0
**>1**	108	20	100	23	18	100	1	0	0.23
**>2**	94	20	100	35	21	100	1.54	0	0.35
**>3**	76	17	85	48	22	95	1.64	0.31	0.33
**>4**	74	16	80	49	22	93	1.57	0.41	0.29
**>6**	57	15	75	63	26	93	2	0.40	0.38
**>8**	50	14	70	68	28	93	2.22	0.44	0.39
**>9**	39	11	55	74	27	90	2.09	0.61	0.28
**>12**	27	9	45	84	33	90	2.85	0.65	0.29
**>18**	23	8	40	87	35	89	3.04	0.69	0.26
**>24**	17	7	35	91	41	89	3.99	0.71	0.26
**>36**	13	5	25	93	38	88	3.56	0.81	0.18
**>48**	5	4	20	99	80	88	22.8	0.81	0.19
**>72**	2	2	10	100	100	86	0	0.9	0.10
**>96**	1	1	5	100	100	86	0	0.95	0.05

AKI; acute kidney injury; Sens, sensitivity; Spec, specificity; PPV, positive predictive value; NPV, negative predictive value, LR+, positive likelihood ratio, LR-, negative likelihood ratio.

Supplemental Table 3. Probability of AKI per subpopulation of RAI and biomarker combination.

RAI	Biomarker	AKI probability		RAI	Biomarker	AKI probability		RAI	Biomarker	AKI probability
RAI Matsuura	Hsp72			RAI Del Toro	Hsp72			mRAI	Hsp72	
-	-	10%		-	-	7%		-	-	6%
-	+	14%		-	+	16%		-	+	9%
+	-	18%		+	-	19%		+	-	21%
+	+	28%		+	+	25%		+	+	24%
RAI Matsuura	NGAL			RAI Del Toro	NGAL			mRAI	NGAL	
-	-	9%		-	-	8%		-	-	8%
-	+	14%		-	+	15%		-	+	7%
+	-	19%		+	-	19%		+	-	20%
+	+	27%		+	+	25%		+	+	36%
RAI Matsuura	TIMP2xIGBFP7			RAI Del Toro	TIMP2xIGBFP7			mRAI	TIMP2xIGBFP7	
-	-	6%		-	-	6%		-	-	2%
-	+	23%		-	+	27%		-	+	25%
+	-	8%		+	-	8%		+	-	14%
+	+	60%		+	+	47%		+	+	58%
RAI Matsuura	CCL14			RAI Del Toro	CCL14			mRAI	CCL14	
-	-	7%		-	-	5%		-	-	5%
-	+	19%		-	+	17%		-	+	11%
+	-	20%		+	-	12%		+	-	14%
+	+	26%		+	+	27%		+	+	33%
RAI Matsuura	KIM-1			RAI Del Toro	KIM-1			mRAI	KIM-1	
-	-	11%		-	-	10%		-	-	8%
-	+	13%		-	+	10%		-	+	10%
+	-	17%		+	-	19%		+	-	26%
+	+	27%		+	+	28%		+	+	29%

Probability of AKI is listed for each subset of patients from decision trees as classified for the outcome of severe Day3 AKI by profile of RAI and biomarker positivity using cut-offs as branch points.
Hsp72 n=122; NGAL n=117; TIMP2xIGFPB7 n=119; CCL14 n=118; KIM-1 n=112 patients.
RAI, renal angina index; mRAI, modified renal angina index; Hsp72, heat shock protein 72; NGAL, neutrophil gelatinase-associated lipocalin; TIMP-2, tissue inhibitor of metalloproteinases-2; IGFBP-7, insulin-like growth factor-binding protein 7; CCL14, C–C motif chemokine ligand 14; KIM-1, kidney injury molecule 1.

Supplemental Table 4. Predictive performance and discrimination for severe Day1-3AKI by the RAI in combination with urinary biomarkers.

	AUC (95% CI)	p-value	AIC	ΔAUC (95% CI)	p-value	IDI	p-value	NRI	p-value
RAI Matsuura (≥10p)	0.63 (0.48-0.78)	0.056	109.7	ref	-	-	-	-	-
Hsp72	0.63 (0.47-0.79)	0.07	103.6	0.04 (-0.06 to 0.14)	0.40	0.0003	0.998	-0.28	0.26
NGAL	0.65 (0.51-0.80)	0.04	94.6	0.04 (-0.04 to 0.12)	0.30	-0.06	0.283	-0.33	0.20
TIMP2 x IGFBP7	0.77 (0.63-0.91)	<0.001	84.2	0.21 (0.04 to 0.37)	**0.01**	-0.17	0.002	0.92	**<0.001**
CCL14	0.64 (0.5-0.78)	0.06	101.8	0.01 (-0.03 to 0.05)	0.52	0.014	0.224	-0.64	**0.014**
KIM-1	0.59 (0.43-0.76)	0.19	100.7	0.006 (-0.04 to 0.05)	0.77	0.005	0.439	-0.27	0.29
RAI Del-Toro-Cisneros (≥8p)	0.71 (0.60-0.82)	0.002	111.6	ref	-	-	-	-	-
Hsp72	0.69 (0.60-0.77)	0.01	104	0.01 (-0.10 to 0.11)	0.88	-0.002	**0.856**	-0.10	0.69
NGAL	0.68 (0.56-0.80)	0.02	95	0.01 (-0.08 a 0.10)	0.88	-0.07	**0.225**	-0.19	0.45
TIMP2 x IGFBP7	0.80 (0.72-0.87)	<0.001	85.1	0.13 (0.01 0.25)	**0.04**	-0.16	**0.001**	0.83	**<0.001**
CCL14	0.68 (0.55-0.81)	0.02	101.9	0.02 (-0.04 0.09)	0.42	0.009	0.431	-0.21	0.42
KIM-1	0.67 (0.54-0.79)	0.02	101.3	0.01 (-0.03 a 0.04)	0.71	0.003	0.508	-0.14	0.58
mRAI (≥8p)	0.76 (0.68-0.83)	<0.001	103.6	ref	-	-	-	-	-
Hsp72	0.72 (0.60-0.84)	<0.001	98.1	0.01 (-0.07 to 0.1)	0.78	0.002	0.868	0.09	0.71
NGAL	0.70 (0.57-0.83)	0.007	91.5	0.02 (-0.06 to 0.1)	0.65	0.035	0.475	-0.007	0.97
TIMP2 x IGFBP7	0.82 (0.72-0.93)	<0.001	84.3	0.1 (-0.01 to 0.23)	0.08	0.141	**0.002**	0.96	**<0.001**
CCL14	0.71 (0.60-0.82)	0.005	98.9	0.0003 (-0.01 to 0.01)	0.97	0.013	0.20	-0.46	0.07
KIM-1	0.68 (0.54-0.82)	0.01	95.8	0.05 (-0.02 to 0.12)	0.17	0.007	0.454	-0.31	0.22

AUC-ROC values were calculated for each prediction model (RAI and biomarker concentrations used as continuous variables) within the measured population and compared using DeLong's method. Integrated Discrimination Improvement (IDI) and Net Reclassification Improvement (NRI) are shown to represent the effect on model prediction after biomarker inclusion. No risk categories were selected for the calculation of NRI. Hsp72 n=122; NGAL n=117; TIMP2xIGFPB7 n=119; CCL14 n=118; KIM-1 n=112 patients. Results are expressed with 95% CI

AUC, area under the receiver operating characteristic; AIC, akaike information criteria; ΔAUC, change in area under the receiver operating characteristic; CI, confidence interval, RAI, renal angina index; mRAI, modified renal angina index; Hsp72, heat shock protein 72; NGAL, neutrophil gelatinase-associated lipocalin; TIMP-2, tissue inhibitor of metalloproteinases-2; IGFBP-7, insulin-like growth factor-binding protein 7; CCL14, C–C motif chemokine ligand 14; KIM-1, Kidney injury molecule 1

Supplemental Table 5. Sensitivity analysis of model performance after multiple imputation by predictive mean matching (PMM) compared with complete-case analysis.

	Complete-case AUC (95% CI)	MI pooled AUC (95% CI)	ΔAUC (95% CI)	p-value
**RAI Matsuura**	0.63 (0.48-0.78)	--	--	--
Hsp72	0.63 (0.47-0.79)	0.67 (0.64-0.7)	0.04 (-0.12 to 0.2)	0.62
NGAL	0.65 (0.51-0.80)	0.67 (0.64-0.7)	0.02 (-0.1 to 0.16)	0.78
TIMP2 x IGFBP7	0.77 (0.63-0.91)	0.78 (0.73-0.80)	0.01 (-0.12 to 0.15)	0.89
CCL14	0.64 (0.5-0.78)	0.64 (0.61-0.68)	0.001 (-0.13 to 0.13)	0.99
KIM-1	0.59 (0.43-0.76)	0.64 (0.6-0.67)	0.05 (-0.1 to 0.2)	0.55
**RAI Del-Toro-Cisneros**	0.71 (0.60-0.82)	--	--	--
Hsp72	0.69 (0.60-0.77)	0.71 (0.68-0.73)	0.02 (-0.09 to 0.13)	0.73
NGAL	0.68 (0.56-0.80)	0.72 (0.69-0.74)	0.04 (-0.08 to 0.16)	0.52
TIMP2 x IGFBP7	0.80 (0.72-0.87)	0.8 (0.78-0.83)	0.001 (-0.1 to 0.1)	0.97
CCL14	0.68 (0.55-0.81)	0.69 (0.66-0.72)	0.01 (-0.13 to 0.15)	0.89
KIM-1	0.67 (0.54-0.79)	0.70 (0.68-0.73)	0.03 (-0.09 to 0.1)	0.63
**mRAI**	0.76 (0.68-0.83)	--	--	--
Hsp72	0.72 (0.60-0.84)	0.75 (0.72-0.77)	0.03 (-0.09 to 0.15)	0.62
NGAL	0.70 (0.57-0.83)	0.73 (0.70-0.76)	0.03 (-0.1 to 0.15)	0.65
TIMP2 x IGFBP7	0.82 (0.72-0.93)	0.83 (0.81-0.85)	-0.01 (-0.09 to 0.11)	0.85
CCL14	0.71 (0.60-0.82)	0.75 (0.73-0.77)	0.04 (-0.07 to 0.15)	0.48
KIM-1	0.68 (0.54-0.82)	0.72 (0.69-0.75)	0.04 (-0.09 to 0.17)	0.57

Area under the ROC curve (AUC) with 95% confidence intervals for complete-case and multiple imputation (MI) analyses. MI performed with predictive mean matching (PMM) for continuous variables (20 imputations). ΔAUC represents the difference between MI and complete-case estimates.

AUC, area under the receiver operating characteristic; MI, multiple imputation; ΔAUC, change in area under the receiver operating characteristic; CI, confidence interval, RAI, renal angina index; mRAI, modified renal angina index; Hsp72, heat shock protein 72; NGAL, neutrophil gelatinase-associated lipocalin; TIMP-2, tissue inhibitor of metalloproteinases-2; IGFBP-7, insulin-like growth factor-binding protein 7; CCL14, C–C motif chemokine ligand 14; KIM-1, Kidney injury molecule 1

Supplemental Figure 1. AKI probability after incorporating biomarkers into the Renal Angina Index Model into decision tree.

The following decision trees (A to O) classify patients based on the likelihood of developing severe acute kidney injury (AKI), using each renal angina index (RAI) in combination with biomarker (UB) positivity or negativity as branch points. The probability of AKI (AKI prob%) is provided for each subset of patients with a specific RAI and biomarker combination.

A) RAI Matsuura and heat-shock-protein 72 (Hsp72):

All patients n=122		
RAI	UB	AKI Probability
-	-	10%
-	+	14%
+	-	18%
+	+	28%

All patients n=122		
	#	Rate
No AKI	104	0.85
AKI	18	0.15

RAI Matsuura <10 n=74		
	#	Rate
No AKI	66	0.89
AKI	8	0.11

RAI Matsuura ≥10 n=48		
	#	Rate
No AKI	38	0.79
AKI	10	0.21

Hsp72 <0.2 n=53		
	#	Rate
No AKI	48	0.90
AKI	5	0.10

Hsp72 ≥0.2 n=21		
	#	Rate
No AKI	18	0.86
AKI	3	0.14

Hsp72 <0.2 n=34		
	#	Rate
No AKI	28	0.82
AKI	6	0.18

Hsp72 ≥0.2 n=14		
	#	Rate
No AKI	10	0.72
AKI	4	0.28

B) RAI Del-Toro-Cisneros and heat-shock-protein 72 (Hsp72):

All patients n=122		
RAI	UB	AKI Probability
-	-	7%
-	+	16%
+	-	19%
+	+	25%

All patients n=122		
	#	Rate
No AKI	104	0.85
AKI	18	0.15

RAI Del-Toro-C <8 n=65		
	#	Rate
No AKI	59	0.90
AKI	6	0.10

RAI Del-Toro-C ≥8 n=57		
	#	Rate
No AKI	45	0.79
AKI	12	0.21

Hsp72 <0.2 n=46		
	#	Rate
No AKI	43	0.93
AKI	3	0.07

Hsp72 ≥0.2 n=19		
	#	Rate
No AKI	16	0.84
AKI	3	0.16

Hsp72 <0.2 n=41		
	#	Rate
No AKI	33	0.81
AKI	8	0.19

Hsp72 ≥0.2 n=16		
	#	Rate
No AKI	12	0.75
AKI	4	0.25

C) Modified RAI (mRAI) and heat-shock-protein 72 (Hsp72):

All patients n=122		
RAI	UB	AKI Probability
-	-	6%
-	+	9%
+	-	21%
+	+	42%

All patients n=122

	#	Rate
No AKI	104	0.85
AKI	18	0.15

mRAI <8 n=72

	#	Rate
No AKI	67	0.93
AKI	5	0.07

mRAI ≥8 n=50

	#	Rate
No AKI	37	0.74
AKI	13	0.26

Hsp72 <0.2 n=49

	#	Rate
No AKI	46	0.94
AKI	3	0.06

Hsp72 ≥0.2 n=23

	#	Rate
No AKI	21	0.91
AKI	2	0.09

Hsp72 <0.2 n=38

	#	Rate
No AKI	30	0.79
AKI	8	0.21

Hsp72 ≥0.2 n=12

	#	Rate
No AKI	7	0.58
AKI	5	0.42

D) RAI Matsuura and Neutrophil gelatinase-associated lipocalin (NGAL)

All patients n=117		
RAI	UB	AKI Probability
-	-	9%
-	+	14%
+	-	19%
+	+	27%

All patients n=117

	#	Rate
No AKI	100	0.85
AKI	17	0.15

RAI Matsuura <10 n=70

	#	Rate
No AKI	63	0.90
AKI	7	0.10

RAI Matsuura ≥10 n=47

	#	Rate
No AKI	37	0.79
AKI	10	0.21

NGAL <152 n=56

	#	Rate
No AKI	51	0.91
AKI	5	0.09

NGAL ≥152 n=14

	#	Rate
No AKI	12	0.86
AKI	2	0.14

NGAL <152 n=32

	#	Rate
No AKI	26	0.81
AKI	6	0.19

NGAL ≥152 n=15

	#	Rate
No AKI	11	0.73
AKI	4	0.27

E) RAI Del-Toro-Cisneros and Neutrophil gelatinase-associated lipocalin (NGAL):

All patients n=117		
RAI	UB	AKI Probability
-	-	8%
-	+	15%
+	-	19%
+	+	25%

All patients n=117

	#	Rate
No AKI	100	0.85
AKI	17	0.15

RAI Del-Toro-C <8 n=64

	#	Rate
No AKI	58	0.91
AKI	6	0.09

NGAL <152 n=51

	#	Rate
No AKI	47	0.92
AKI	4	0.08

NGAL ≥152 n=13

	#	Rate
No AKI	11	0.85
AKI	2	0.15

RAI Del-Toro-C ≥8 n=53

	#	Rate
No AKI	42	0.79
AKI	11	0.21

NGAL <152 n=37

	#	Rate
No AKI	30	0.81
AKI	7	0.19

NGAL ≥152 n=16

	#	Rate
No AKI	12	0.75
AKI	4	0.25

F) Modified RAI (mRAI) and Neutrophil gelatinase-associated lipocalin (NGAL):

All patients n=117		
RAI	UB	AKI Probability
-	-	8%
-	+	7%
+	-	20%
+	+	36%

All patients n=117

	#	Rate
No AKI	100	0.85
AKI	17	0.15

mRAI <8 n=69

	#	Rate
No AKI	64	93
AKI	5	0.07

NGAL <152 n=54

	#	Rate
No AKI	50	0.92
AKI	4	0.08

NGAL ≥152 n=15

	#	Rate
No AKI	14	0.93
AKI	1	0.07

mRAI ≥8 n=48

	#	Rate
No AKI	36	0.75
AKI	12	0.25

NGAL <152 n=34

	#	Rate
No AKI	27	0.80
AKI	7	0.20

NGAL ≥152 n=14

	#	Rate
No AKI	9	0.64
AKI	5	0.36

G) RAI Matsuura and Tissue Inhibitor of Metalloproteinases-2 (TIMP2) / Insulin-like Growth Factor-Binding Protein 7 (IGFBP7):

All patients n=119		
RAI	UB	AKI Probability
-	-	6%
-	+	23%
+	-	8%
+	+	60%

All patients n=119		
	#	Rate
No AKI	102	0.86
AKI	17	0.14

RAI Matsuura <10 n=71		
	#	Rate
No AKI	63	0.89
AKI	8	0.11

RAI Matsuura ≥10 n=48		
	#	Rate
No AKI	39	0.81
AKI	9	0.19

TIMP-2 x IGFBP7 <2 n=53		
	#	Rate
No AKI	50	0.94
AKI	3	0.06

TIMP-2 x IGFBP7 ≥2 n=18		
	#	Rate
No AKI	13	0.72
AKI	5	0.23

TIMP-2 x IGFBP7 <2 n=38		
	#	Rate
No AKI	35	0.92
AKI	3	0.08

TIMP-2 x IGFBP7 ≥2 n=10		
	#	Rate
No AKI	4	0.4
AKI	6	0.6

H) RAI Del-Toro-Cisneros and Tissue Inhibitor of Metalloproteinases-2 (TIMP2) / Insulin-like Growth Factor-Binding Protein 7 (IGFBP7):

All patients n=119		
RAI	UB	AKI Probability
-	-	6%
-	+	27%
+	-	8%
+	+	47%

All patients n=119		
	#	Rate
No AKI	102	0.86
AKI	17	0.14

RAI Del-Toro-C <8 n=63		
	#	Rate
No AKI	57	0.90
AKI	6	0.10

RAI Del-Toro-C ≥8 n=56		
	#	Rate
No AKI	45	0.80
AKI	11	0.20

TIMP-2 x IGFBP7 <2 n=52		
	#	Rate
No AKI	49	0.94
AKI	3	0.06

TIMP-2 x IGFBP7 ≥2 n=11		
	#	Rate
No AKI	8	0.73
AKI	3	0.27

TIMP-2 x IGFBP7 <2 n=39		
	#	Rate
No AKI	36	0.92
AKI	3	0.08

TIMP-2 x IGFBP7 ≥2 n=17		
	#	Rate
No AKI	9	0.53
AKI	8	0.47

I) Modified RAI (mRAI) and Tissue Inhibitor of Metalloproteinases-2 (TIMP2) /Insulin-like Growth Factor-Binding Protein 7 (IGFBP7):

All patients n=119		
RAI	UB	AKI Probability
-	-	2%
-	+	25%
+	-	14%
+	+	58%

All patients n=119		
	#	Rate
No AKI	102	0.86
AKI	17	0.14

mRAI <8 n=70		
	#	Rate
No AKI	65	0.93
AKI	5	0.07

mRAI ≥8 n=49		
	#	Rate
No AKI	37	0.76
AKI	12	0.24

TIMP-2 x IGFBP7 <2 n=54		
	#	Rate
No AKI	53	0.98
AKI	1	0.02

TIMP-2 x IGFBP7 ≥2 n=16		
	#	Rate
No AKI	12	0.75
AKI	4	0.25

TIMP-2 x IGFBP7 <2 n=37		
	#	Rate
No AKI	32	0.86
AKI	5	0.14

TIMP-2 x IGFBP7 ≥2 n=12		
	#	Rate
No AKI	5	0.42
AKI	7	0.58

J) RAI Matsuura and Chemokine (C-C motif) Ligand 14 (CCL14):

All patients n=118		
RAI	UB	AKI Probability
-	-	7%
-	+	19%
+	-	20%
+	+	26%

All patients n=118		
	#	Rate
No AKI	101	0.86
AKI	17	0.14

RAI Matsuura <10 n=71		
	#	Rate
No AKI	63	0.89
AKI	8	0.11

RAI Matsuura ≥10 n=47		
	#	Rate
No AKI	38	0.81
AKI	9	0.19

CCL14 <250 n=44		
	#	Rate
No AKI	41	0.93
AKI	3	0.07

CCL14 ≥250 n=27		
	#	Rate
No AKI	22	0.81
AKI	5	0.19

CCL14 <250 n=20		
	#	Rate
No AKI	18	0.90
AKI	2	0.20

CCL14 ≥250 n=27		
	#	Rate
No AKI	20	0.74
AKI	7	0.26

K) RAI Del-Toro-Cisneros and Chemokine (C-C motif) Ligand 14 (CCL14):

All patients n=118		
RAI	UB	AKI Probability
-	-	5%
-	+	17%
+	-	12%
+	+	27%

All patients n=118		
	#	Rate
No AKI	101	0.86
AKI	17	0.14

RAI Del-Toro-C <8 n=63		
	#	Rate
No AKI	57	0.90
AKI	6	0.10

RAI Del-Toro-C ≥8 n=55		
	#	Rate
No AKI	44	0.80
AKI	11	0.20

CCL14 <250 n=39		
	#	Rate
No AKI	37	0.95
AKI	2	0.05

CCL14 ≥250 n=24		
	#	Rate
No AKI	20	0.83
AKI	4	0.17

CCL14 <250 n=25		
	#	Rate
No AKI	22	0.88
AKI	3	0.12

CCL14 ≥250 n=30		
	#	Rate
No AKI	22	0.73
AKI	8	0.27

L) Modified RAI (mRAI) and Chemokine (C-C motif) Ligand 14 (CCL14):

All patients n=118		
RAI	UB	AKI Probability
-	-	5%
-	+	11%
+	-	14%
+	+	33%

All patients n=118		
	#	Rate
No AKI	101	0.86
AKI	17	0.14

mRAI <8 n=69		
	#	Rate
No AKI	57	0.90
AKI	5	0.10

mRAI ≥8 n=49		
	#	Rate
No AKI	44	0.80
AKI	12	0.20

CCL14 <250 n=42		
	#	Rate
No AKI	40	0.95
AKI	2	0.05

CCL14 ≥250 n=27		
	#	Rate
No AKI	24	0.89
AKI	3	0.11

CCL14 <250 n=22		
	#	Rate
No AKI	19	0.86
AKI	3	0.14

CCL14 ≥250 n=27		
	#	Rate
No AKI	18	0.66
AKI	9	0.33

M) RAI Matsuura and Kidney Injury Molecule-1 (KIM1):

All patients n=112		
RAI	UB	AKI Probability
-	-	11%
-	+	13%
+	-	17%
+	+	27%

All patients n=112		
	#	Rate
No AKI	94	0.84
AKI	18	0.16

RAI Matsuura <10 n=67		
	#	Rate
No AKI	59	0.88
AKI	8	0.12

RAI Matsuura ≥10 n=45		
	#	Rate
No AKI	35	0.78
AKI	10	0.22

KIM-1 <1.4 n=35		
	#	Rate
No AKI	31	0.89
AKI	4	0.11

KIM-1 ≥1.4 n=32		
	#	Rate
No AKI	28	0.87
AKI	4	0.13

KIM-1 <1.4 n=23		
	#	Rate
No AKI	19	0.83
AKI	4	0.17

KIM-1 ≥1.4 n=22		
	#	Rate
No AKI	16	0.73
AKI	6	0.27

N) RAI Del-Toro-Cisneros and Kidney Injury Molecule-1 (KIM1):

All patients n=112		
RAI	UB	AKI Probability
-	-	10%
-	+	10%
+	-	19%
+	+	28%

All patients n=112		
	#	Rate
No AKI	94	0.84
AKI	18	0.16

RAI Del-Toro-C <8 n=60		
	#	Rate
No AKI	54	0.90
AKI	6	0.10

RAI Del-Toro-C ≥8 n=52		
	#	Rate
No AKI	40	0.77
AKI	12	0.23

KIM-1 <1.4 n=31		
	#	Rate
No AKI	28	0.90
AKI	3	0.10

KIM-1 ≥1.4 n=29		
	#	Rate
No AKI	26	0.90
AKI	3	0.10

KIM-1 <1.4 n=27		
	#	Rate
No AKI	22	0.81
AKI	5	0.19

KIM-1 ≥1.4 n=25		
	#	Rate
No AKI	18	0.72
AKI	7	0.28

O) Modified RAI (mRAI) and Kidney Injury Molecule-1 (KIM1):

All patients n=112		
RAI	UB	AKI Probability
-	-	8%
-	+	10%
+	-	26%
+	+	29%

All patients n=112		
	#	Rate
No AKI	94	0.84
AKI	18	0.16

mRAI <8 n=65		
	#	Rate
No AKI	60	0.92
AKI	5	0.08

mRAI ≥8 n=47		
	#	Rate
No AKI	34	0.72
AKI	13	0.28

KIM-1 <1.4 n=35		
	#	Rate
No AKI	33	0.94
AKI	2	0.06

KIM-1 ≥1.4 n=30		
	#	Rate
No AKI	27	0.90
AKI	3	0.10

KIM-1 <1.4 n=23		
	#	Rate
No AKI	17	0.74
AKI	6	0.26

KIM-1 ≥1.4 n=24		
	#	Rate
No AKI	17	0.71
AKI	7	0.29
